# Role of self-efficacy and social support in short-term recovery after total hip replacement: a prospective cohort study

**DOI:** 10.1186/s12955-017-0649-1

**Published:** 2017-04-11

**Authors:** Espen Andreas Brembo, Heidi Kapstad, Sandra Van Dulmen, Hilde Eide

**Affiliations:** 1 Faculty of Health and Social Sciences, University College of Southeast Norway, P.O Box 7053, 3007, Papirbredden - Drammen kunnskapspark Grønland 58, Drammen, 3045 Norway; 2 Department of Behavioral Sciences in Medicine, Institute of Basic Medical Sciences, Faculty of Medicine, University of Oslo, Sognsvannsveien 9, Oslo, 0372 Norway; 3 NIVEL (Netherlands institute for health services research), Otterstraat 118-124, Utrecht, 3513 CR The Netherlands; 4 Department of Primary and Community Care, Radboud University Medical Center, Geert Grooteplein noord 21, Nijmegen, 6525 EZ The Netherlands

**Keywords:** Osteoarthritis, Psychosocial predictors, Total hip replacement, Social support, Self-efficacy, WOMAC

## Abstract

**Background:**

Despite the overall success of total hip replacement (THR) in patients with symptomatic osteoarthritis (OA), up to one-quarter of patients report suboptimal recovery. The aim of this study was to determine whether social support and general self-efficacy predict variability in short-term recovery in a Norwegian cohort.

**Methods:**

We performed secondary analysis of a prospective multicenter study of 223 patients who underwent THR for OA in 2003–2004. The total score of the Western Ontario and McMaster Universities Osteoarthritis Index (WOMAC) at 3 months after surgery was used as the recovery variable. We measured self-efficacy using the General Self-Efficacy Scale (GSES) and social support with the Social Provisions Scale (SPS). Preoperative and postoperative scores were compared using Wilcoxon tests. The Mann–Whitney *U* test compared scores between groups that differed in gender and age. Spearman’s rho correlation coefficients were used to evaluate associations between selected predictor variables and the recovery variable. We performed univariate and multiple linear regression analyses to identify independent variables and their ability to predict short-term recovery after THR.

**Results:**

The median preoperative WOMAC score was 58.3 before and 23.9 after surgery. The mean absolute change was 31.9 (standard deviation [SD] 17.0) and the mean relative change was 54.8% (SD 26.6). Older age, female gender, higher educational level, number of comorbidities, baseline WOMAC score, self-efficacy, and three of six individual provisions correlated significantly with short-term recovery after THR and predicted the variability in recovery in the univariate regression model. In multiple regression models, baseline WOMAC was the most consistent predictor of short-term recovery: a higher preoperative WOMAC score predicted worse short-term recovery (β = 0.44 [0.29, 0.59]). Higher self-efficacy predicted better recovery (β = −0.44 [−0.87, −0.02]). Reliable alliance was a significant predictor of improved recovery (β = −1.40 [−2.81, 0.01]).

**Conclusions:**

OA patients’ general self-efficacy and the expectation of others’ tangible assistance predict recovery after THR. Researchers and clinicians should target these psychosocial factors together with the patients and their families to improve the quality of care and surgical outcomes.

**Electronic supplementary material:**

The online version of this article (doi:10.1186/s12955-017-0649-1) contains supplementary material, which is available to authorized users.

## Background

Osteoarthritis (OA) is the most common form of arthritis and involves inflammation and major structural changes of the joint, which cause pain and functional disability. Pain, often in association with exercise, is a hallmark symptom and has a considerable effect on the ability to perform activities of daily living [1]. Moderate to severe OA is the most common indication for total hip replacement (THR). Although the prevalence and incidence may differ between populations, OA is considered to be a worldwide disease [2, 3].

According to recommendations, THR is indicated when the patient’s OA-related functional limitations and pain levels are refractory to pharmacological and nonpharmacological treatment modalities [4, 5]. THR is a cost-effective treatment for hip OA and offers relief of pain and improved function and quality of life [6]. In Norway, 8,099 primary hip replacements were performed in 2014, about 80% (6,369) of which were for patients with primary hip OA [7].

Studies demonstrate good clinical outcomes [8, 9], but some patients fail to recover optimally from THR [10, 11]. Although THR generally resolves pain, function usually remains substantially suboptimal. For example, 24 months following total joint arthroplasty, patients with low preoperative function are five times more likely to require assistance from another person for their activities of daily living compared with those with high preoperative function. A systematic review reported that 7–23% of the patients undergoing THR experienced suboptimal outcomes 3 months to 5 years after the procedure [12]. Hawker et al. [11] reported that nearly half of their study participants had poor outcomes such as pain and function following total joint replacement; these were mostly elderly patients with additional comorbidities.

In general, patients with lower baseline function seem to experience greater pain and worse function compared with those with higher baseline function [13, 14]. This is called the “better in, better out” concept; that is, the better the condition of the patient coming into the hospital, the better and more quickly he/she leaves the hospital [15]. Therefore, improving each patient’s health status before surgery should produce better outcomes at an individual level. Unfit patients might be advised to postpone surgery to optimize preoperative functional status, whereas other patients might benefit from undergoing surgery earlier in the course of functional decline [16].

Few studies have identified the psychosocial predictors associated with recovery following THR. In this study, we investigated the role of patients’ social support and general self-efficacy because OA causes substantial physical disability and has considerable psychosocial consequences that can affect the patient’s ability to maintain or improve physical health [17]. Self-efficacy refers to a person’s confidence in his/her ability to successfully execute and accomplish a specific task [18]; a more generalized sense of self-efficacy is conceptualized as “a global confidence in one’s coping ability across a wide range of demanding or novel situations” [19]. Social support can be defined as those resources in a person’s environment that enable that person to deal with life’s physical and psychological stresses. For example, a patient may be extremely disabled but may be able to maintain a high quality of life because of effective social support. Surgery, such as THR, magnifies the need for short-term support. The effect of social support on health is a complex phenomenon to investigate, and varies with the specific dimensions of support as well as with the exact outcome being considered [20]. The role of social support as a factor predicting postoperative outcomes in OA joint replacement patients has not been extensively studied. Various definitions and conceptualizations, and the use of different outcome measures contribute to the lack of conclusive evidence.

In this study, we used general measures of self-efficacy and social support. The overall aim was to determine whether perceived social support and general self-efficacy contribute to the variability in short-term postoperative recovery in a sample of OA patients who have undergone THR. A secondary aim was to determine whether recovery, social support, and self-efficacy differ according to gender, age group, or number of comorbidities.

## Methods

### Study design and sample

**Fig. 1:**
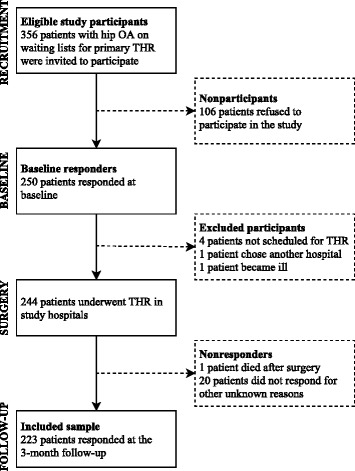
Sample flowchart

This study comprised a secondary analysis of longitudinal data from research conducted by one of the authors (HK), who prospectively explored changes in pain and health status among patients with hip or knee OA who underwent joint replacement [21–24]. In the present study, we analyzed the data for patients with hip OA who completed the study package of questionnaires preoperatively and at 3 months following primary THR (Fig. 1). Adult patients aged >18 years who were placed on the waiting list for a primary THR were recruited consecutively in 2003–2004 at six hospitals in three Norwegian counties.

### Questionnaires

Patients completed a package of questionnaires that obtained sample characteristics, including gender, age, marital status, cohabitation, number of children, educational level, employment status, comorbidity, and number of years with hip pain and reduced mobility (before the decision to undergo THR). At baseline and at 3 months after surgery, they also reported overall satisfaction with life on a 7-point Likert scale, and pain and mobility levels on a 5-point Likert scale. Patients also completed the Western Ontario and McMaster Universities Osteoarthritis Index (WOMAC) [25], the General Perceived Self-Efficacy Scale (GSES) [26], and the Social Provisions Scale (SPS) [27].

### WOMAC

WOMAC is a widely used disease-specific questionnaire developed to study health status in patients with hip or knee OA. It has a multidimensional scale comprising 24 items grouped into three dimensions: pain (five items), stiffness (two items), and physical function (17 items). We used the 3.0 Likert version with five response categories for each item representing different degrees of intensity (none, mild, moderate, severe, or extreme) and scored from 0 to 4 [25]. The total WOMAC score was chosen as an appropriate outcome measure of recovery after THR and was calculated by adding the aggregate scores for pain, stiffness, and physical function. The data were standardized to scales with values from 0 to 100, where 0 represents the best health status and 100 the worst health status. Missing data were handled according to the user’s manual [25]. Previous research has shown WOMAC to be reliable, valid, and sensitive to changes in the health status of patients with hip or knee OA [28].

### GSES

We measured patients’ self-efficacy using the GSES with 10 items [26]. The GSES is widely used, reliable, homogeneous, and unidimensional [29, 30]. All items have the following response format: 1 = not at all true, 2 = hardly true, 3 = moderately true, and 4 = exactly true. The total GSES score is calculated by summing the item scores, and ranges between 10 (lowest GSES) and 40 (highest GSES). We calculated the sum score in this study for subjects with no more than three items missing [31]. Examples of items in the GSES are “I can always manage to solve difficult problems if I try hard enough” and “It is easy for me to stick to my aims and accomplish my goals”.

### SPS

Perceived social support was assessed using the revised SPS [27]. This 24-item instrument asks respondents to rate the degree to which their social relationships currently are supplying each of six relational provisions [32]: guidance, reliable alliance, reassurance of worth, attachment, social integration, and opportunity for nurturance. Each provision is assessed by four items: two describing the presence and two the absence of the provision. Respondents indicate on 4-point scales the extent to which each statement describes their current social relationships. For scoring purposes, the negative items are reversed and summed together with the positive items to form a score for each social provision, which gives a minimum score of 4 and a maximum score of 16. An aggregated social support score is also calculated with a minimum score of 24 points and a maximum score of 96. A high score indicates a high degree of perceived social support. Internal consistency (alpha coefficient) for the SPS has been reported to range from 0.85 to 0.92 across a variety of populations and from 0.64 to 0.76 for the individual subscales [33]. Evidence supports the reliability and validity of the SPS [27].

### Statistical methods

We used IBM SPSS Statistics for Windows version 23.0 [34] to organize and analyze the data. Descriptive statistics were used to estimate the data for sample characteristics. We compared groups of responders and nonresponders using Pearson’s chi-squared test, independent samples *t* test, or Mann–Whitney *U* test, where applicable. Preoperative and postoperative WOMAC total and dimension scores were compared using nonparametric related-samples (Wilcoxon) tests. The Mann–Whitney *U* test was used to compare scores between gender and age groups. We assessed internal consistency reliability of the questionnaires using Cronbach’s coefficient alpha. Pearson and Spearman rank correlation coefficients were used to identify the variables for inclusion in the regression analyses based on associations between selected predictor variables and the primary measure of recovery at 3 months after THR, as appropriate. We included predictors based on the availability of data and on our theoretical hypothesis about possible relationships relating to the aim of the study: age, gender, cohabitation, number of children, education level, work status, number of comorbidities, years with hip pain, years with mobility problems, overall satisfaction with life and baseline scores (including WOMAC, SPS, and GSES). Linear regression models were used to study the associations between the predictors and the recovery variable WOMAC total. We applied the following steps after a thorough evaluation of the theoretical assumptions relevant to linear regression.

1. Predictor variables that correlated with the recovery variable (α = 0.10) were included into a univariate linear regression model. This step identified how well each variable predicted recovery after THR without controlling for any confounding factors.
2. The next step was to proceed with a multiple linear regression model. We included all predictors with a significant association with the recovery variable (α = 0.05) in the initial model. Residual plots were controlled.
3. To identify the best predictive model of recovery after THR, we used a backward elimination procedure. For each step in this stepwise procedure, we evaluated each β value and its 95% confidence interval. Nonsignificant predictors were omitted sequentially from the model until all remaining variables were statistically significant in explaining the variance in post-THR recovery.

## Results

### Sample characteristics

We invited 356 patients with hip OA to participate; 250 (70%) accepted and responded at baseline, and 223 (89%) patients returned the questionnaire at the 3-month follow-up. Twenty-seven patients did not respond; four of whom did not undergo THR, one chose another hospital, one became ill and one died after surgery (Fig. 1). Because the questionnaires were mailed to eligible participants, we do not know the reasons why the remaining twenty patients did not respond at follow-up. We compared differences in gender and age among baseline responders and patients who refused to participate [see Additional file 1]. There were no differences in gender (*χ*2 (1) = 0.27, p = 0.61). However, nonparticipants were older (73.6 years (SD = 8.9)) than baseline responders (69.3 (SD = 9.6), p = < 0.01). Table 1 presents the baseline characteristics of responders and nonresponders.

**Table 1:** Sample characteristics at baseline for responders 3 months after surgery, and nonresponders

	Responders	Nonresponders	*P*-value
N	223	27	
Age (mean ± standard deviation)	69.3 ± 9.8	69.1 ± 8.5	0.92
Female gender	159 (71.3)	20 (74.1)	0.83
Marital status			0.49
Married	149 (66.8)	17 (63.0)	
Widowed	38 (17.0)	5 (18.5)	
Divorced/separated	25 (11.2)	5 (18.5)	
Single	11 (5.0)	-	
Living with someone	156 (70.0)	19 (70.4)	1.0
Having children	194 (92.8)	27 (100)	0.23
Educational level			0.09
Primary school	54 (24.4)	13 (48.1)	
Secondary school	94 (42.5)	10 (37.0)	
University <4 years	41 (18.6)	2 (7.4)	
University ≥4 years	32 (14.5)	2 (7.4)	
Employment			0.60
Retired	144 (64.6)	17 (63.0)	
Full- or part-time work	35 (15.7)	2 (7.4)	
Sick leave	18 (8.1)	3 (11.1)	
Disability pension	26 (11.6)	5 (18.5)	
Number of comorbidities			0.11
1	91 (40.8)	17 (63.0)	
2	55 (24.7)	4 (16.0)	
3	29 (13)	4 (16.0)	
>3	10 (4.5)	–	
Missing data	38 (17)	2 (7.4)	

Number (%) unless otherwise stated

The responders and nonresponders had similar characteristics in our sample. Responders included 159 women and 64 men with a mean age of 69 years; 21% were younger than 60 years, and 29% were older than 75 years. The youngest patient was 41 years old at the time of surgery and the oldest was 91 years. Most reported being married and not living alone. One-third had a higher educational level. Most (78%) had 1–3 comorbid conditions, such as cardiovascular, gastrointestinal, pulmonary, or psychiatric conditions, cancer, skin diseases, or diabetes mellitus. Thirty-eight patients (17%) did not respond to this question, and we do not know whether this indicated no comorbidity or whether the question was left blank for other reasons. The patients had experienced hip pain for an average of about 6 years. At the time of the baseline assessment, 108 patients (48%) reported severe pain, and 20 patients (14%) reported extreme pain. When asked about mobility, 157 patients reported having severe (52%) or extreme (19%) problems; 61% reported being somewhat or less satisfied with life.

### Comparison of responders and nonresponders’ baseline WOMAC, SPS, and GSES scores

The responder and nonresponder groups were compared to account for any nonresponse bias. The two groups did not differ on any of the scales [see Additional file 2].

### Short-term recovery following THR

Table 2 provides the baseline and postoperative scores for WOMAC and its subscales. Normality testing of the recovery variable showed a moderately skewed distribution with a positive skewness value of 0.96 (standard error of skewness = 0.17). Assessment of internal consistency reliability of the WOMAC baseline scores suggested satisfactory results, with Cronbach’s alpha values of 0.78, 0.69, 0.93, and 0.94 for the subscales, pain, stiffness, physical function, and total score, respectively. Patients reported a mean WOMAC total score of 57.7 points at the baseline and 25.6 points at 3 months after THR, yielding a mean absolute change of 31.9 points (standard deviation [SD] 17.0) and a mean relative change of 54.8% (SD 26.6). Women had significantly higher mean scores than men both at the baseline and at 3 months (60.0 vs 51.9 [*P* < 0.001] and 27.0 vs 22.4 [*P* = 0.023]). There was no difference in the mean absolute change between men and women (32.9 vs 30.0 points [*P* = 0.41]).

**Table 2:** WOMAC, SPS and GSES scores at the baseline and 3 months after THR

	Baseline mean (SD)	Quartiles 1^st^, 2^nd^, 3^rd^	α	3 months post-THR mean (SD)	Quartiles 1^st^, 2^nd^, 3^rd^	α	*P*-value
	*N* = 218			*N* = 218			
WOMAC total	57.7 (14.5)	49, 58.3, 67.7	0.94	25.6 (16.1)	13.5, 23.9, 34.4	0.96	<0.001
Pain	56.3 (17.5)	45, 55, 69.7	0.78	16.8 (16.6)	5, 10, 25	0.88	<0.001
Stiffness	60.8 (17.8)	50, 62.5, 75	0.69	31.5 (17.2)	25, 25, 43.8	0.74	<0.001
Physical function	57.6 (15.2)	48.5, 58.8, 68.7	0.93	27.7 (17.3)	14.7, 25, 38.2	0.95	<0.001
	*N* = 220			*N* = 219			
SPS	86.3 (8.2)	82.6, 89, 92	0.85	86.1 (8.9)	82, 88, 93	0.86	0.96
Guidance	15.0 (2.0)	15, 16, 16	0.79	14.9 (2.2)	15, 16, 16	0.73	0.70
Reliable alliance	15.2 (1.6)	15, 16, 16	0.51	15.3 (1.7)	16, 16, 16	0.67	0.28
Attachment	14.9 (1.7)	14, 16, 16	0.56	14.7 (1.9)	14, 16, 16	0.62	0.22
Social integration	14.4 (1.7)	13, 15, 16	0.53	14.3 (1.8)	14, 15, 16	0.61	0.67
Reassurance of worth	14.6 (1.8)	14, 15, 16	0.67	14.7 (1.7)	14, 15, 16	0.69	0.28
Opportunity for nurturance	12.3 (2.9)	10, 13, 15	0.68	12.2 (3.1)	10, 13, 15	0.72	0.41
	*N* = 217			*N* = 216			
GSES	30.9 (5.2)	28, 30, 34,7	0.92	31.6 (4.7)	29, 31, 35	0.91	0.018

WOMAC (0–100): high score indicates worse recovery. SPS (24–96 [total score], 4–16 [subscores]: high score indicates a greater degree of perceived support. GSES (10–40): high score indicates a high level of self-efficacy

A comparison of patients younger and older than 70 years (median as the cut point) showed no differences in baseline scores between the two groups (57.5 vs 57.9 points [*P* = 0.78]). However, the older patients (*n* = 109) had a worse score at 3 months (23.3 vs 28.0 points [*P* = 0.059]). Younger patients had higher absolute change scores compared with older patients (34.4 vs 29.4 points [*P* = 0.02]). Patients who reported having two or more comorbidities (median as the cut point) had significantly higher mean scores (29.1) at 3 months compared with those reporting one comorbid condition (22.8 [*P* = 0.032]). Accordingly, these patients reported lower absolute change scores (28.8) than did patients with one comorbid condition (35.3 [*P* = 0.014]).

### Change in social support and general self-efficacy

Normality testing of the baseline and postoperative SPS scores showed highly skewed distributions with negative skewness values of −1.27 and −1.74, respectively. As seen in Table 2, the internal consistency of the baseline scores was good for the aggregated social support score (0.85) and excellent for the GSES score (0.92). Patients’ perceived social support remained stable across all dimensions and did not change significantly from the baseline to the 3-month follow-up. The same trend was observed for self-efficacy, although a small but significant absolute change was observed (0.6 points [*P* = 0.02]). A comparison between men and women showed no significant differences for baseline social support (86.1 vs 86.7 [*P* = 0.53]). However, women reported significantly lower self-efficacy scores than men (30.6 vs 31.8 [*P* = 0.044]). No significant difference in absolute change scores between men and women was observed.

A comparison between patients younger and older than 70 years showed significantly higher social support at the baseline in younger patients (88.1 vs 84.6 [*P* = 0.001]). This pattern was also evident for all SPS subscales except for reliable alliance (15.3 vs 15.2 [*P* = 0.38]) and reassurance of worth (14.8 vs 14.3 [*P* = 0.064]). The scores for the significant subscales were: guidance (15.3 vs 14.7 [*P* = 0.006]); attachment (15.2 vs 14.7 [*P* = 0.007]); social integration (14.7 vs 14.0 [*P* = 0.006]); and opportunity for nurturance (12.8 vs 11.9 [*P* = 0.05]). The same trend appeared for baseline self-efficacy (31.8 vs 30.1 [*P* = 0.009]). There was no significant difference according to age in absolute change scores. The baseline and absolute changes in social support and self-efficacy scores did not differ according to comorbidity groups.

### Prediction of short-term recovery after THR

Regression diagnostic analyses revealed an acceptable distribution of the residuals associated with the outcome variable.

#### Step 1. Univariate analysis

The following predictor variables correlated significantly with the recovery variable WOMAC total [see Additional file 3] and were included in the univariate regression analysis: age (Spearman rank-order coefficient [r_s_] = 0.15 [*P* = 0.03]), female gender (r_s_ = 0.15 [*P* = 0.03]), educational level (r_s_ = 0.17 [*P* = 0.01]), cohabitation (r_s_ = −0.12 [P = 0.08]), number of comorbidities (r_s_ = 0.16 [*P* = 0.04]), baseline WOMAC (r_s_ = 0.37 [*P* < 0.001]), baseline GSES (r_s_ = −0.18 [*P* = 0.01]), baseline SPS total (r_s_ = −0.13 [*P* = 0.06]), reliable alliance (r_s_ = −0.13 [*P* = 0.06]), social integration (r_s_ = −0.12 [*P* = 0.07]), and reassurance of worth (r_s_ = −0.14 [*P* = 0.04]). No significant correlations were found with the remaining predictors considered: number of children, full- or part-time work, number of years with pain and mobility problems, guidance, attachment, and opportunity for nurturance. Table 3 shows that the baseline WOMAC scores were the most significant independent predictors of short-term recovery with an R^2^ of 0.15. Patient characteristics such as older age, lower educational level, and increased number of comorbidities were associated with worse recovery. Being female was borderline significant and predicted worse recovery. Cohabitation did not reach statistical significance, but the coefficient indicates that living alone predicted better recovery. Greater baseline self-efficacy and perceived social support predicted better recovery. Of the six relational provisions measured by the SPS, the presence of reliable alliances, social integration, and reassurance of worth appeared to independently predict better recovery.

**Table 3:** Univariate linear regression analysis

Predictors	WOMAC total score (0–100)						
	β	95% CI		Std. Error	*P*-value	R^2^	N
		Lower	Upper				
Age	0.26	0.05	0.45	0.11	0.02	0.03	218
Female gender	4.56	−0.13	9.25	2.38	0.06	0.02	218
Living alone	−3.86	−8.60	0.89	2.41	0.11	0.01	218
Higher education	−5.30	−9.85	−0.74	2.31	0.02	0.02	218
Comorbidity	2.79	0.47	5.10	1.17	0.02	0.03	180
Baseline WOMAC total (0–100)	0.46	0.30	0.58	0.07	<0.001	0.15	213
Self-efficacy (10–40)	−0.52	−0.93	−0.11	0.21	0.01	0.03	212
Social support (16–96)	−0.26	−0.52	−0.003	0.13	0.05	0.02	215
Reliable alliance (4–16)	−2.13	−3.48	−0.78	0.69	0.002	0.04	214
Social integration (4–16)	−1.26	−2.54	0.02	0.65	0.05	0.02	212
Reassurance of worth (4–16)	−1.41	−2.63	−0.19	0.62	0.02	0.02	211

#### Steps 2 and 3: Multiple linear regression analysis

When we included predictors from the univariate analysis into a multiple regression model, the model explained about 29% of the variance in recovery 3 months after THR. Following the elimination procedure, gender, educational level, aggregate SPS score, social integration, and reassurance of worth did not contribute statistically to recovery after THR and were therefore omitted from the model. By contrast, self-efficacy and reliable alliance appeared to be significant predictors even after adjusting for age, number of comorbidities, and preoperative WOMAC. The final linear regression model explained 28.5% of the variance in short-term recovery (Table 4).

**Table 4:** Multiple regression model

Predictors	WOMAC total score (0–100)				
	β	95% CI		Std. Error	*P*-value
		Lower	Upper		
*Constant*	7.66	−24.81	40.13	16.45	0.64
Age	0.35	0.13	0.57	0.21	0.002
Comorbidity	2.12	0.06	4.19	1.05	0.04
Baseline WOMAC total (0–100)	0.44	0.29	0.59	0.08	<0.001
Self-efficacy (10–40)	−0.44	−0.87	−0.02	0.22	0.04
Social support					
Reliable alliance (4–16)	−1.40	−2.81	0.01	0.71	0.05

R^2^ = 0.285, *N* = 172

## Discussion

To our knowledge, this is the first prospective study to evaluate whether general self-efficacy and perceived social support predict short-term recovery following THR in patients with hip OA. The data used in this study were gathered more than 10 years ago; however, the patient care pathways have not changed to any appreciable extent, and the results should still be relevant.

### Role of self-efficacy and social support

Higher preoperative levels of reliable alliances and general self-efficacy tended to independently predict better recovery from THR, even after adjusting for age, number of comorbidities, and preoperative WOMAC score. These are clinically relevant findings because these factors are considered as constructs that can be modified through behavioral interventions and tailoring of evidence-based treatment plans. A person’s attitudes toward behavior change, self-efficacy, and social influences are modeled as vital factors within the integrated model for explaining motivational and behavioral change (I-Change Model) [35].

Neither social support nor general self-efficacy seemed to change as a consequence of undergoing THR. This result suggests that perceived social support is an indicator of stable social relationships and environment, and that general self-efficacy is a personal trait measure in this context. Self-efficacy is not considered to be a personality trait but rather a situation-specific construct [36, 37]. However, in contrast to other domain-specific instruments [38], the GSES maps self-efficacy as the global confidence in one’s coping ability across a wide range of demanding or novel situations. Generalized positive beliefs of self-efficacy serve as a resource factor that buffers against distress experiences. Weak self-efficacy beliefs make a person vulnerable to distressing experiences by causing the person to be permanently worried, have weak expectancies of task-specific competence, interpret physiological arousal as an indicator of anxiety, regard achievement feedback as social evaluations of personal value, and feel more responsible for failure than for success [19, 26]. Further research is needed to determine the role of generalized self-efficacy beliefs in the self-management of hip OA. Treatment strategies that incorporate psychological factors initiated in the early phases of the disease continuum [39] and that include an explicit effort to increase patients’ self-efficacy beliefs and supportive networks, will increase the probability that patients will enter surgery with more confidence and ultimately experience better recovery [40].

Most patients undergoing THR are discharged directly to their home. It is therefore not surprising that assurance of tangible assistance seems to predict outcomes after surgery. This quality of social support is usually obtained from family members [33] and has been reported to be a significant predictor of recovery after joint replacement surgery. One study, in which social support was measured by the Medical Outcomes Study Social Support Scale, found that worse postoperative WOMAC function scores were predicted by less tangible support, depression, and decreased problem-solving coping [41]. Escobar et al. took a different approach to measure this dimension of social support [42], and asked the responders whether they would have assistance during recovery after total knee replacement (TKR). Their analysis indicated that patients who expected assistance had better scores at 6 months after surgery in the three WOMAC domains and in four of the eight Short Form Health Survey (SF-36) domains. A cruder measure of social support can be obtained by dichotomizing patients who report being married or living with someone. Patients undergoing THR or TKR who were either married or living with someone were defined as having more social support than those who were not married or lived alone. The presence of social support was associated with improved SF-36 bodily pain and physical function outcomes [43]. McHugh, Campbell, and Luker [44] investigated the predictive factors of recovery after THR in a prospective study involving 206 patients. Social support, as measured by the ENRICHD Social Support Instrument, did not predict recovery at 6 or 12 months after surgery, where recovery was defined as gains in the total physical score dimension of the SF-36 questionnaire.

We found no other studies that have used the GSES or SPS questionnaires to identify predictors of recovery after THR; however, some studies have used other questionnaires or methods to measure these constructs. A Dutch study evaluated the contributions of preoperative and short-term postoperative self-efficacy in predicting long-term outcomes measured 6 months after THR or TKR [45]. The Self-Efficacy for Rehabilitation Outcome Scale was used to assess self-efficacy preoperatively and at 6 weeks after surgery. Preoperative self-efficacy was a significant predictor only of long-term postoperative walking speed; higher self-efficacy was associated with faster walking speed. Short-term postoperative self-efficacy was a significant predictor of the postoperative SF-36 subscales physical functioning and mental health, and of walking speed; higher self-efficacy was associated with a better long-term outcome. In another study of patients undergoing TKR, preoperative self-efficacy, as measured by the Pain Self-Efficacy Scale, was a significant predictor of functional ability but not pain 1 year after surgery [46]. These results were included in a systematic review [47] that concluded that preoperative self-efficacy was the least consistent predictor of functional outcomes, whereas postoperative self-efficacy was more consistently associated with recovery outcomes such as longer distance ambulation, exercise repetition and frequency, walking speed, and disability. However, as noted by the authors of that review, no statistical synthesis was possible because of the number of, and variation in, the measures used (both for predictor and outcome variables) and the different timing of the assessment of self-efficacy and outcome.

Clearly, different ways of conceptualizing and measuring self-efficacy and social support and the use of different outcome variables complicate comparisons with existing studies and the ability to draw firm conclusions about the predictive capacity of these constructs. Nevertheless, our findings suggest that self-efficacy and social support deserve more attention in future research and patient care planning.

### Strengths and limitations

One strength of this study is that we used validated questionnaires to measure self-efficacy and social support. Power calculations were conducted to ensure that the planned sample size was large enough to detect clinically significant changes. The procedure is explained elsewhere [21]. Our findings supplement the limited literature on the role of social support and self-efficacy as predictors of recovery after THR. Importantly, our results can be used for comparisons in future studies. Except for the assessment of comorbidities, we achieved a low rate of missing data, with fewer than seven patients failing to complete the preoperative or postoperative WOMAC, SPS, and GSES assessments.

The study also has some limitations. The age difference between participants and nonparticipants may represent a selection bias in this study, and thus affect the representativeness of the sample. As also reported in the literature [48, 49], increasing age predicted worse recovery in this study. We can therefore assume that this does not directly impede the validity of our findings. The number of comorbidities is a significant risk factor for recovery after THR. However, 17% of the participants did not respond to the question about this, possibly because there was no response category to indicate zero comorbidities. This high percentage of missing data limits the validity of the findings, and our data should be confirmed in a new study with validated methods to assess comorbidity [50]. We note that there was no significant difference in the WOMAC, GSES, or SPS scores between the groups with and without missing data on comorbidity. As reported in the literature, previous joint surgeries can negatively affect outcomes following THR [44, 51]. However, this information was not available in the dataset, and we therefore could not control for this possible confounder variable. For the subscale reliable alliance, the internal consistency (Cronbach’s alpha of 0.51) may be questioned. One explanation might be the negatively skewed distribution of the data (skewness value of −2.25). Inspection of the unusual cases led us to believe that some respondents misinterpreted the negatively worded statements or may have responded uncritically similarly across the whole subscale because their responses did not correspond with the equivalent positively worded statements. These results should be interpreted with caution because of this low alpha score.

## Conclusions

Increasing age, preoperative WOMAC score, and number of comorbidities are factors associated with worse recovery after THR. By contrast, the presence of reliable alliances and higher general self-efficacy are associated with better recovery. For clinicians, these findings may provide indicators of the need for relevant interventions to be introduced at an early time point. Further studies should use valid measurements and test tailored interventions to enhance the outcomes of patients at risk of suboptimal recovery after THR.

## Additional files

Additional file 1: Age and gender differences in baseline responders and nonparticipants. Table presenting a comparison of age and gender in patients who accepted to participate vs patients who refused to participate. (DOCX 12 kb)

Additional file 2: Baseline differences among responders and nonresponders. Table presenting baseline scores of WOMAC, SPS and GSES among responders and nonresponders and a comparison of group differences. (DOCX 13 kb)

Additional file 3: Spearman rank-order coefficient correlations, rs (*p*-value) between baseline predictors and the recovery variable. Table displaying correlations between baseline predictors and the recovery variable, WOMAC total. (DOCX 13 kb)
