# Supplementary material for: Role of self-efficacy and social support in short-term recovery after total hip replacement: a prospective cohort study
Source: Health Qual Life Outcomes. 2017 Apr 11;15:68. doi: 10.1186/s12955-017-0649-1 (PMC5387328; doi:10.1186/s12955-017-0649-1)
Supplement: Supplementary file 1 — Age and gender differences in baseline responders and nonparticipants. Table presenting a comparison of age and gender in patients who accepted to participate vs patients who refused to participate. (DOCX 12 kb) [file 12955_2017_649_MOESM1_ESM.docx]

Additional file 1: Age and gender differences in baseline responders and nonparticipants

|  | Baseline responders | Nonparticipants | *P*-value |
| --- | --- | --- | --- |
| N | 250 | 106 |  |
| Age (mean ± standard deviation) | 69.3 ± 9.6 | 73.6 ± 8.9 | <0.001 |
| **Gender** |  |  | 0.61 |
| Females | 179 (71.6) | 73 (68.9) |  |
| Males | 71 (28.4) | 33 (31.1) |  |

Number (%) unless otherwise stated
