# Supplementary material for: Role of self-efficacy and social support in short-term recovery after total hip replacement: a prospective cohort study
Source: Health Qual Life Outcomes. 2017 Apr 11;15:68. doi: 10.1186/s12955-017-0649-1 (PMC5387328; doi:10.1186/s12955-017-0649-1)
Supplement: Supplementary file 2 — Baseline differences among responders and nonresponders. Table presenting baseline scores of WOMAC, SPS and GSES among responders and nonresponders and a comparison of group differences. (DOCX 13 kb) [file 12955_2017_649_MOESM2_ESM.docx]

Additional file 2: Baseline differences among responders and nonresponders

|  | **Responders** | | **Nonresponders** | | **p-value** |
| --- | --- | --- | --- | --- | --- |
|  | **Mean (SD)** | **Quartiles**  1^st^, 2^nd^, 3^rd^ | **Mean (SD)** | **Quartiles**  1^st^, 2^nd^, 3^rd^ |  |
|  | *N*=218 | | *N*=27 | |  |
| **WOMAC total** | 57.7 (14.5) | 49, 58.3, 67.7 | 58.9 (18.3) | 38.5, 61.5, 71.9 | 0.40 |
| Pain | 56.3 (17.5) | 45, 55, 69.7 | 58.1 (19.2) | 40, 60, 75 | 0.41 |
| Stiffness | 60.8 (17.8) | 50, 62.5, 75 | 62.5 (19.0) | 50, 62.5, 75 | 0.62 |
| Physical function | 57.6 (15.2) | 48.5, 58.8, 68.7 | 58.6 (19.1) | 38.2, 62.5, 75 | 0.45 |
|  | *N* = 220 | | *N* = 25 | |  |
| **SPS** | 86.3 (8.2) | 82.6, 89, 92 | 85.2 (9.0) | 79.5, 88.7, 92,0 | 0.59 |
| Guidance | 15.0 (2.0) | 15, 16, 16 | 14.4 (2.7) | 13, 16, 16 | 0.49 |
| Reliable alliance | 15.2 (1.6) | 15, 16, 16 | 15.3 (1.4) | 15, 16, 16 | 0.89 |
| Attachment | 14.9 (1.7) | 14, 16, 16 | 15.3 (1.2) | 14.5, 16, 16 | 0.46 |
| Social integration | 14.4 (1.7) | 13, 15, 16 | 13.9 (1.9) | 13, 14, 15,5 | 0.20 |
| Reassurance of worth | 14.6 (1.8) | 14, 15, 16 | 14.2 (2.0) | 12.5, 15, 16 | 0.51 |
| Opportunity for nurturance | 12.3 (2.9) | 10, 13, 15 | 12.2 (3.0) | 9.5, 12, 16 | 0.74 |
|  | *N* = 217 | | *N* = 25 | |  |
| **GSES** | 30.9 (5.2) | 28, 30, 34,7 | 30.7 (5.4) | 27.4, 30, 35.5 | 0.77 |

WOMAC (0-100 [total and sub-scores]): high score indicates worse recovery. SPS (24-96 [total score], 4-16 [sub-scores]): high score indicates a greater degree of perceived support. GSES [10-40]: high score indicates a high level of self-efficacy. Mann Whitney U test compares responders and non-responders (alpha=.05).
