# Supplementary material for: Role of self-efficacy and social support in short-term recovery after total hip replacement: a prospective cohort study
Source: Health Qual Life Outcomes. 2017 Apr 11;15:68. doi: 10.1186/s12955-017-0649-1 (PMC5387328; doi:10.1186/s12955-017-0649-1)
Supplement: Supplementary file 3 — Spearman rank-order coefficient correlations, rs (p-value) between baseline predictors and the recovery variable. Table displaying correlations between baseline predictors and the recovery variable, WOMAC total. (DOCX 13 kb) [file 12955_2017_649_MOESM3_ESM.docx]

Additional file 3: Spearman rank-order coefficient correlations, r_s_ (p-value) between baseline predictors and the recovery variable

| **Baseline predictors** | **WOMAC total** |
| --- | --- |
| Age | 0.15 (0.03) |
| Female gender | 0.15 (0.03) |
| Cohabitation | –0.12 (0.08) |
| Number of children | 0.06 (0.39) |
| Education level | –0.17 (0.01) |
| Full or part time work | –0.11 (0.11) |
| Comorbidities | 0.16 (0.04) |
| Years with hip pain | –0.06 (0.42) |
| Years with mobility problems | 0.02 (0.83) |
| Overall satisfaction with life | 0.10 (0.13) |
| Baseline WOMAC total | 0.37 (<0.001) |
| Baseline GSES | –0.18 (0.01) |
| Baseline SPS total | –0.13 (0.06) |
| Guidance | –0.09 (0.20) |
| Reliable alliance | –0.13 (0.06) |
| Attachment | 0.01 (0.88) |
| Social integration | –0.12 (0.07) |
| Reassurance of worth | –0.14 (0.04) |
| Opportunity for nurturance | –0.03 (0.68) |
